# Heterogeneity of cognitive progression and clinical predictors in Parkinson’s disease–subjective cognitive decline

**DOI:** 10.1007/s00415-024-12808-0

**Published:** 2025-03-05

**Authors:** Jon Rodríguez-Antigüedad, Saül Martínez-Horta, Arnau Puig-Davi, Andrea Horta-Barba, Javier Pagonabarraga, Teresa de Deus Fonticoba, Silvia Jesús, Marina Cosgaya, Juan García Caldentey, María Asunción Ávila-Rivera, Nuria Caballol, Inés Legarda, Jorge Hernández Vara, Iria Cabo, Lydia López Manzanares, Isabel González Aramburu, Víctor Gómez Mayordomo, Jessica González Ardura, Julio Dotor García-Soto, Carmen Borrué, Berta Solano Vila, María Álvarez Sauco, Lydia Vela, Sonia Escalante, Esther Cubo, Zebenzui Mendoza, Isabel Pareés, Pilar Sánchez Alonso, María G. Alonso Losada, Nuria López Ariztegui, Itziar Gastón, Javier Ruíz Martínez, María Teresa Buongiorno, Carlos Ordás, Caridad Valero, Víctor Puente, Mónica Kurtis, Marta Blázquez Estrada, Pablo Martínez-Martín, Pablo Mir, Diego Santos-García, COPPADIS Study Group, A. D. Adarmes, M. Almeria, M. G. Alonso Losada, A. Alonso Cánovas, F. Alonso Frech, R. Alonso Redondo, I. Álvarez, M. Álvarez Sauco, A. Aneiros Díaz, S. Arnáiz, S. Arribas, A. Ascunce Vidondo, M. Aguilar, M. A. Ávila, N. Bernardo Lambrich, H. Bejr-Kasem, M. Blázquez Estrada, M. Botí, C. Borrue, M. T. Buongiorno, C. Cabello González, I. Cabo López, N. Caballol, A. Cámara Lorenzo, H. Canfield Medina, E. Carabajal Pendón, F. Carrillo, F. J. Carrillo Padilla, E. Casas, M. J. Catalán, P. Clavero, A. Cortina Fernández, M. Cosgaya, A. Cots Foraster, A. Crespo Cuevas, E. Cubo, T. de Deus Fonticoba, O. de Fábregues-Boixar, M. Díez-Fairen, J. Dotor García-Soto, E. Erro, S. Escalante, E. Estelrich Peyret, N. Fernández Guillán, P. Gámez, M. Gallego, J. García Caldentey, C. García Campos, C. García Díez, J. M. García Moreno, I. Gastón, M. P. Gómez Garre, V. Gómez Mayordomo, J. González Aloy, I. González-Aramburu, J. González Ardura, B. González García, M. J. González Palmás, G. R. González Toledo, A. Golpe Díaz, M. Grau Solá, G. Guardia, J. Hernández Vara, A. Horta-Barba, D. Idoate Calderón, J. Infante, S. Jesús, J. Kulisevsky, M. Kurtis, C. Labandeira, M. A. Labrador, F. Lacruz, M. Lage Castro, S. Lastres Gómez, I. Legarda, N. López Ariztegui, L. M. López Díaz, D. López Domínguez, L. López Manzanares, B. López Seoane, S. Lucas del Pozo, Y. Macías, M. Mata, G. Martí Andres, M. J. Martí, J. C. Martínez Castrillo, P. Martinez-Martin, D. McAfee, M. T. Meitín, Z. Mendoza Plasencia, M. Menéndez González, C. Méndez del Barrio, P. Mir, J. Miranda Santiago, M. I. Morales Casado, A. Moreno Diéguez, I. Muro García, V. Nogueira, A. Novo Amado, S. Novo Ponte, C. Ordás, J. Pagonabarraga, I. Pareés, B. Pascual-Sedano, P. Pastor, A. Pérez Fuertes, R. Pérez Noguera, A. Planas-Ballvé, L. Planellas, M. A. Prats, C. Prieto Jurczynska, V. Puente, M. Pueyo Morlans, A. Puig Daví, N. Redondo Rafales, L. Rodríguez Méndez, A. B. Rodríguez Pérez, F. Roldán, M. Ruíz De Arcos, J. Ruíz Martínez, P. Sánchez Alonso, M. Sánchez-Carpintero, G. Sánchez Díez, A. Sánchez Rodríguez, P. Santacruz, D. Santos García, J. C. Segundo Rodríguez, M. Seijo, M. Sierra Peña, B. Solano Vila, E. Suárez Castro, J. P. Tartari, C. Valero, L. Vargas, L. Vela, C. Villanueva, B. Vives, Jaime Kulisevsky

**Affiliations:** 1 Medicine Department, Universitat Autònoma de Barcelona (UAB), Barcelona, Spain; 2 Movement Disorders Unit, Neurology Department, Hospital de la Santa Creu i Sant Pau, Mas Casanovas 90, 08041 Barcelona, Spain; 3 Institut d´Investigacions Biomèdiques-Sant Pau (IIB-Sant Pau), Barcelona, Spain; 4 Centro de Investigación Biomédica en Red-Enfermedades Neurodegenerativas (CIBERNED), Madrid, Spain; 5 Neurology Department, Complejo Hospitalario Universitario de Ferrol (CHUF), A Coruña, Spain; 6 Movement Disorders Unit, Neurology and Clinical Neurophysiology Department, Instituto de Biomedicina de Sevilla, Hospital Universitario Virgen del Rocío/CSIC, Universidad de Sevilla, Seville, Spain; 7 Neurology Department, Hospital Clínic de Barcelona, Barcelona, Spain; 8 Centro Neurológico Oms 42, Neurology Department, Palma, Spain; 9 Neurology Department, Consorci Sanitari Integral, Hospital Moisés Broggi Sant Joan Despí, Barcelona, Spain; 10 Neurology Department, Hospital Universitario Son Espases, Palma, Spain; 11 Neurology Department, Hospital Universitario Vall d´Hebron, Barcelona, Spain; 12 Neurology Department, Complejo Hospitalario Universitario de Pontevedra (CHOP), Pontevedra, Spain; 13 Neurology Department, Hospital Universitario La Princesa, Madrid, Spain; 14 Neurology Department, Hospital Universitario Marqués de Valdecilla-IDIVAL, Santander, Spain; 15 Neurology Department, Institute of Neuroscience, Vithas Madrid La Milagrosa University Hospital, Vithas Hospital Group, Madrid, Spain; 16 Neurology Department, Hospital de Cabueñes, Gijón, Spain; 17 Neurology Department, Hospital Universitario Virgen Macarena, Seville, Spain; 18 Neurology Department, Hospital Infanta Sofía, Madrid, Spain; 19 Neurology Department, Institut d’Assistència Sanitària (IAS), Institut Català de la Salut, Girona, Spain; 20 Neurology Department, Hospital General Universitario de Elche, Elche, Spain; 21 Neurology Department, Fundación Hospital de Alcorcón, Madrid, Spain; 22 Neurology Department, Hospital de Tortosa Verge de la Cinta (HTVC), Tortosa, Tarragona Spain; 23 Neurology Department, Complejo Asistencial Universitario de Burgos, Burgos, Spain; 24 Neurology Department, Hospital Universitario de Canarias, San Cristóbal de la Laguna, Santa Cruz de Tenerife, Spain; 25 Neurology Department, Hospital Universitario Ramón y Cajal, IRYCIS, Madrid, Spain; 26 Neurology Department, Hospital Universitario Puerta de Hierro, Madrid, Spain; 27 Neurology Department, Hospital Álvaro Cunqueiro, Complejo Hospitalario Universitario de Vigo (CHUVI), Vigo, Spain; 28 Neurology Department, Complejo Hospitalario de Toledo, Toledo, Spain; 29 Neurology Department, Complejo Hospitalario de Navarra, Pamplona, Spain; 30 Neurology Department, Hospital Universitario Donostia, Donostia, Spain; 31 Neurology Department, Hospital Universitari Mutua de Terrassa, Terrassa, Barcelona Spain; 32 Neurology Department, Hospital Rey Juan Carlos, Madrid, Spain; 33 Neurology Department, Hospital Arnau de Vilanova, Valencia, Spain; 34 Neurology Department, Hospital del Mar, Barcelona, Spain; 35 Neurology Department, Hospital Ruber Internacional, Madrid, Spain; 36 Neurology Department, Hospital Universitario Central de Asturias, Oviedo, Spain; 37 Neurology Department, Complejo Hospitalario Universitario de A Coruña (CHUAC), INIBIC, A Coruña, Spain; 38 Fundación Española de Ayuda a La Investigación en Enfermedades Neurodegenerativas y/o de Origen Genético, Oleiros, Spain

**Keywords:** Parkinson’s Disease, Cognitive complaints, Cognitive decline, PD-Subjective Cognitive Decline, REM-sleep behavior disorder

## Abstract

**Background:**

Parkinson’s Disease (PD)-associated subjective cognitive decline (PDSCD) is defined as cognitive complaints without objective cognitive impairment. Based on most studies, it is associated with a greater risk of cognitive decline and may represent a prodromal stage of cognitive impairment.

**Methods:**

The main objectives are to identify cognitive progression patterns and clinical predictors of worse cognitive decline within a large PD-SCD cohort with a 4-year followup. All patients belong to the prospective observational multicenter study COPPADIS.

**Results:**

A total of 198 PD-SCD subjects were analyzed. Mean age was 60.9, mean disease duration 5.2, and mean PD-Cognitive Rating Scale (PD-CRS) 97.6. Subjects were classified as Progressors if their Reliable Change Index was ≤ − 1.64 at year 4, and as non-Progressors if it was > − 1.64 (− 1.64 corresponded to − 16 on the PD-CRS). Progressors had significantly higher age, Movement Disorders Society-Unified PD Rating Scale (MDS-UPDRS) III, levodopa equivalent daily-dose, Non-Motor Symptom Scale total score, memory-related cognitive complaints, and prevalence of REM-sleep behavior disorder (RBD) at baseline. A linear mixed-effects model showed divergent cognitive trajectories between Progressors and non-Progressors (estimate = − 26.8; p < 0.001), with no differences in motor trajectories. In the binary regression model, age (OR = 1.09; p = 0.001), MDS-UPDRS III (OR = 1.05, p = 0.008), and RBD (OR = 2.55, p = 0.010) at baseline were independent predictors of cognitive progression.

**Conclusions:**

Subjects with PD-SCD do not consistently show cognitive decline, but rather exhibit a heterogeneous progression. Age, MDS-UPDRS III and RBD significantly increase the risk of a more aggressive cognitive phenotype. Future research on biomarkers will help explore additional cognitive predictors in PD-SCD.

## Introduction

Cognitive impairment is a frequent and disabling non-motor symptom (NMS) that can occur at any point along Parkinson’s disease (PD) [1]. The spectrum of cognitive impairment in PD ranges from mild cognitive impairment (PD–MCI) to dementia (PDD), and its pattern of progression exhibits a significant heterogeneity between individuals [2, 3]. From a neuropsychological perspective, the most prominent characteristics of cognitive impairment in PD involve fronto-striatal-dependent features mostly in the form of attentional, working memory, planning and set-shifting deficits, whereas memory, language, and visuospatial compromise is more commonly observed in the transition to or in already established PDD [3, 4].

In recent decades, there has been growing interest in the concept of subjective cognitive decline in PD (PD–SCD), which is believed to represent an intermediate state between normal cognition (PD–NC) and PD–MCI [5–7]. The term PD–SCD has been borrowed from the field of Alzheimer’s disease and is defined as a self-reported decline in cognitive capacities with normal performance in cognitive tests in patients already diagnosed with PD [8]. Due to the lack of consensus on the assessment tools and diagnostic criteria for cognitive complaints and PD–SCD, data on its prevalence in persons with PD (PwP) are inconsistent, with studies reporting prevalences ranging from 15 to 83% [5, 9, 10]. A recent meta-analysis estimated a prevalence of 36% for cognitive complaints, and growing evidence suggests that PD–SCD is a precursor to future cognitive impairment, increasing its risk by 2.7 times [6, 9, 10].

While the majority of studies corroborate the heightened risk of developing cognitive impairment in PD–SCD, some of them have not evidenced this association [11, 12]. Similarly to other symptoms in PD, cognitive complaints may not invariably predict cognitive decline. Identifying diverse progression patterns and their predictors has significant implications for both clinical and research settings, as this population in the prodromal stage of cognitive impairment represents an ideal target for clinical trials focused on disease-modifying interventions.

The objectives of the present study are to describe *data-driven* patterns of cognitive progression using a PD-validated scale in a large longitudinal cohort, and to identify baseline clinical predictors of a more pronounced cognitive decline. For this purpose, we longitudinally analyzed a cognitively unimpaired PD cohort with PD–SCD, over a 4-year period.

## Methods

### Participants and study design

Participants were recruited between January 2016 and November 2017 from 35 centers across Spain for the COhort of Patients with PArkinson’s Disease in Spain, 2015 (COPPADIS). This is a prospective 5-year follow-up study designed to investigate the natural progression of PD. Selection criteria included individuals aged 35–75 years, diagnosed with PD according to the UK PD Brain Bank criteria, without dementia, and without advanced therapies for PD such as infusion therapies or deep brain stimulation. This project received approval from the ethics committee in Galicia, Spain (2014/534; 02/DEC/2014). All participants voluntarily provided a written informed consent to participate. Additional study details can be found in the original publication [13]. For the present study, we selected PwP with normal cognitive performance as assessed with the Parkinson’s Disease–Cognitive Rating Scale total score (PD–CRS total score > 81) and with cognitive complaints as assessed with the NMS Scale (NMSS) domain 5 [4, 14]. All the included subjects had completed motor and cognitive assessments of the 4-year follow-up visit.

To assess the presence of cognitive complaints we used the NMSS domain 5, which includes three rater-administered questions: item 16 “Does the patient have problems sustaining concentration during activities?”, item 17 “Does the patient forget things that he/she has been told a short time ago or events that happened in the last few days?” and item 18 “Does the patient forget to do things?”. Participants were categorized as PD–SCD if the score in NMSS domain 5 was ≥ 1.

### Assessments

Demographic data, motor assessments [Movement Disorders Society–Unified PD Rating Scale (MDS–UPDRS) III, and Hoehn and Yahr scale (H&Y)], cognitive assessment (PD–CRS), neuropsychiatric and other NMS scales [Beck Depression Inventory II (BDI-II), and NMSS] were collected from all participants at baseline, 2 years, and 4 years. NMSS was used to assess the presence of different NMS: domain 1 for cardiovascular autonomic symptoms; domain 3 for depression, anxiety, and apathy; domain 5 for memory and concentration problems; domain 6 for gastrointestinal autonomic symptoms; and domain 7 for urinary autonomic symptoms [15]. The presence of visual hallucinations was determined using the item 13 of the NMSS (“Does the patient indicate that he/she sees thing that are not there?”). A score > 0 was considered indicative of this symptom. The presence of hyposmia was considered when a score > 0 was obtained for the item 28 of the NMSS (“Does the patient report a change in ability to taste or smell?”). REM–sleep behavior disorder (RBD) was determined through a clinical interview conducted by a movement disorders neurologist.

Subjects were categorized into progression groups using the Reliable Change Index (RCI) for the PD–CRS total score. This index evaluates whether the change over time can be attributed to measurement error or not [16]. To calculate the RCI we applied the following formula: RCI = (X_2_–X_1_)/S_diff_, where X_1_ is the patient’s PD–CRS score at baseline, X_2_ is the patient’s PD–CRS score at 4 years, and S_diff_ is the standard error of the difference between the test scores. Similar to previous literature, we employed a 90% confidence level [17]. Subjects were labeled as PD–SCD Progressors if their RCI was ≤ −1.64, and as non-progressors if their RCI > −1.64.

### Statistical analysis

Descriptive statistics for demographic and clinical features of each PD–SCD subgroup (progressors and non-progressors), as well as differences between them were explored using an independent two-tailed *t* test for continuous variables and the *χ*2 test for categorical variables. Continuous variables are presented as means and standard deviations (SD), while categorical variables are presented as numbers and percentages. Linear mixed-effects model (LME) with the lme4 package was conducted to model the longitudinal trajectories of cognition and motor symptoms over time within each PD–SCD subgroup. Binary logistic regression was used to explore the independent association between the variables of interest and the PD–SCD subgroup (backward stepwise method). All the variables used for the LME and logistic regression model adjustments are specified in the *Results* section. The analyses were conducted using IBM–SPSS software (v26) and R (v4.3.3). A two-tailed *p* value of < 0.05 was considered significant for all tests.

### Data availability

The protocol and the statistical analysis plan are available on request. Deidentified participant data are not available for legal and ethical reasons.

## Results

A total of 198 subjects with PD–SCD from a cohort of 326 cognitively unimpaired individuals were included for analysis. The mean age was 60.9 ± 8.6 years, and 41.1% were women. The sample consisted of subjects in the early stage of PD (mean disease duration of 5.2 ± 4 years, mean H&Y stage of 1.9 ± 0.6 and mean MDS–UPDRS III of 21.2 ± 11.1) with a PD–CRS total score significantly above the cutoff of ≤ 81 for PD–MCI (mean PD–CRS total score of 97.6 ± 10.5). The mean BDI-II score (8.7 ± 7.1) indicated minimal depressive symptoms [18]. The mean difference between PD–CRS total scores at the 4-year follow-up and baseline was −5.5 ± 14.8. All demographics and baseline characteristics are reported in Table 1.

**Table 1:** Demographics and baseline characteristics of the PD–SCD cohort

PD–SCD cohort	*n* = 198
Demographics	
Age, years	60.9 (8.6)
Biological sex, women (%)	81–41.1%
Education level, years	11.9 (4.1)
Motor symptoms	
Disease duration, years	5.2 (4)
H&Y	1.9 (0.6)
MDS–UPDRS III	21.2 (11.1)
LEDD, mg	560 (379)
Cognitive/ neuropsychiatric	
PD–CRS frontal subcortical score	69.7 (10.2)
PD–CRS posterior cortical score	28 (3.3)
PD–CRS total score	97.6 (10.5)
Visual hallucinations (%)	60–30.3%
BDI–II total score	8.7 (7.1)
NMSS domain 3—mood	9.5 (11.9)
Other NMS	
RBD (%)	82–41.4%
Hyposmia (%)	141–71.2%
NMSS domain 1—cardiovascular	1.5 (2.4)
NMSS domain 6—gastrointestinal	3.9 (4.7)
NMSS domain 7—urinary	8.3 (7.9)
NMSS total score	52.1 (38.5)

Continuous variables are presented as mean (standard deviation), and categorical variables are presented as number–percentage. *PD–SCD* PD–subjective cognitive decline; *H&Y* Hoehn and Yahr scale; *MDS–UPDRS* Movement Disorders Society–Unified PD Rating Scale; *LEDD* Levodopa equivalent daily dose; *NMSS* non-motor symptom scale; *PD–CRS* PD–Cognitive Rating Scale; *BDI–II* beck depression inventory II; *RBD* REM–sleep behavior disorder

We calculated the RCI to categorize subjects into two distinct groups based on their progression in the PD–CRS. For this purpose, we determined internal reliability using Cronbach's alpha (0.782 for the 9 items of the PD–CRS). Subjects were labeled as Progressors if their RCI was ≤ −1.64, whereas those with an RCI > −1.64 were labeled as non-Progressors. The threshold of −1.64 corresponded to a loss of 16 points in the PD–CRS total score at the 4-year follow-up. At baseline, Progressors showed a significantly higher age [t(196) = 3.3; *p* = 0.001], MDS–UPDRS III [t(193) = 2.4; *p* = 0.018], LEDD [t(196) = 3; *p* = 0.003], and NMSS total score [t(195) = 2.2; *p* = 0.030], and higher prevalence of RBD (*p* = 0.002). Memory complaints were more severe in the Progressors group as measured by NMSS domain 5 items 17 [t(195) = 2.1; *p* = 0.036] and 18 [t(195) = 2.3; *p* = 0.024], while no significant differences were found in concentration/attention complaints, as measured by NMSS domain 5 item 16. The proportion of subjects diagnosed with PD–MCI level I at year 4 was markedly higher in the progressors group (87% vs 9.2%, *p* < 0.001), despite no differences in PD–CRS total score at baseline. Baseline characteristics of both subgroups are summarized in Table 2.

**Table 2:** Demographics and baseline characteristics of PD–SCD progressors and non-progressors

	Progressors (n = 46)	non-progressors (n = 152)	*p* value
Demographics			
Age, years	64.5 (7.1)	59.8 (8.8)	**0.001**
Biological sex, women (%)	20–43.5%	62–40.8%	0.746
Education level, years	11.1 (3.8)	12.2 (4.2)	0.122
Motor symptoms			
Disease duration, years	5.6 (4.9)	5.1 (3.7)	0.508
H&Y	2 (0.6)	1.9 (0.5)	0.060
MDS–UPDRS III	24.6 (11.5)	20.1 (10.9)	**0.018**
LEDD, mg	703 (438)	517 (350)	**0.003**
Cognitive/ neuropsychiatric			
NMSS domain 5 Item 16	2.4 (2.8)	2.2 (2.6)	0.611
NMSS domain 5 Item 17	2.4 (2.9)	1.6 (2.1)	**0.036**
NMSS domain 5 Item 18	1.8 (2.7)	1 (1.9)	**0.024**
PD–CRS frontal subcortical score	67.4 (8.2)	70.4 (10.6)	0.079
PD–CRS posterior cortical score	27.6 (3.9)	28.1 (3.1)	0.391
PD–CRS total score	95 (9.1)	98.4 (10.8)	0.054
Visual hallucinations (%)	18–39.1%	42–27.6%	0.137
BDI–II total score	8.8 (6.2)	8.7 (7.4)	0.914
NMSS domain 3—mood	11.7 (11.6)	8.8 (12)	0.157
Other NMS			
RBD (%)	28–60.9%	54–35.5%	**0.002**
Hyposmia (%)	36–78.3%	105–69.1%	0.228
NMSS domain 1—cardiovascular	2.2 (3)	1.3 (2.2)	0.050
NMSS domain 6—gastrointestinal	5 (5.4)	3.5 (4.4)	0.064
NMSS domain 7—urinary	10 (8.3)	7.8 (7.7)	0.095
NMSS total score	62.9 (42.4)	48.8 (36.7)	**0.030**

*p* values < 0.05 are highlighted in bold

Continuous variables are presented as mean (standard deviation), and categorical variables are presented as number–percentage. *H&Y* Hoehn and Yahr scale; *MDS–UPDRS* Movement Disorders Society–Unified PD Rating Scale; *LEDD* Levodopa equivalent daily dose; *NMSS* Non-Motor Symptom Scale; *PD–CRS* PD–Cognitive Rating Scale; *BDI-II* Beck Depression Inventory II; RBD: REM–sleep behavior disorder

**Fig. 1:**
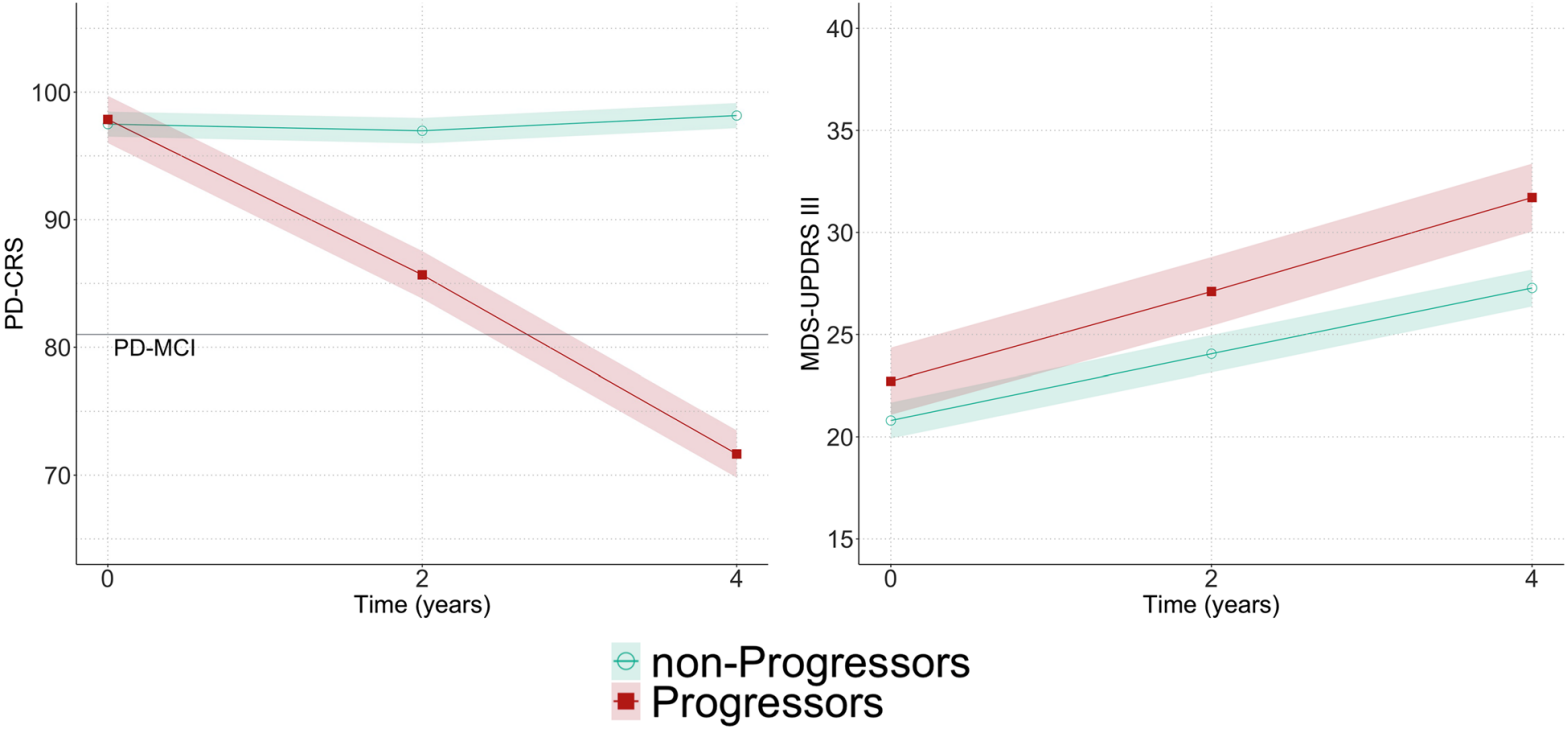
Predictions of the LME model for the longitudinal trajectory of PD–CRS (left) and MDS–UPDRS III (right) in PD–SCD progressors and non-progressors

To explore the size of the PD–CRS trajectory differences between the two subgroups, an LME model was employed. The fixed effects included age, disease duration, MDS–UPDRS III, LEDD, and NMSS total score. Random effects were incorporated to account for potential variations between individuals. The model predicted a marked worsening in PD–CRS total score at 4 years in progressors [estimate (*β*) −26.9, standard error (SE) 1.6, *p* < 0.001] compared to non-progressors (Fig. 1, left; Table 3). A subsequent LME model to examine the trajectories of MDS–UPDRS III after adjusting for age, disease duration, LEDD and NMSS total score as fixed effects, did not show statistically significant differences between Progressors (*β* 2.5, SE 1.9, *p* = 0.179) and non-progressors (Fig. 1, right; Table 3).

**Table 3:** Results of the LME model for PD–CRS and MDS–UPDRS (reference group: non-progressors)

	PD–CRS			MDS–UPDRS		
	Estimate	Standard error	*p* value	Estimate	Standard error	*p* value
Age	−0.58	0.09	** < 0.001**	−0.03	0.08	0.675
Disease duration	0.31	0.26	0.230	0.15	0.21	0.476
LEDD	−0.004	0.003	0.176	0.01	0.002	** < 0.001**
NMSS total score	−0.01	0.02	0.493	0.07	0.02	** < 0.001**
MDS–UPDRS III	−0.01	0.02	0.141	–	–	–
Progressors x Timepoint 1 (year 2)	−11.66	1.60	** < 0.001**	1.13	1.89	0.551
Progressors x Timepoint 2 (year 4)	−26.87	1.57	** < 0.001**	2.51	1.87	0.179

*p* values < 0.05 are highlighted in bold

*PD–CRS* PD–cognitive rating scale; *MDS–UPDRS* Movement Disorders Society–Unified PD Rating Scale; *LEDD* Levodopa equivalent daily dose

Finally, we incorporated into a binary regression model (progressors vs non-progressors) those baseline variables that had shown significant association in the univariate analysis and had no multicollinearity between them, as well as other variables of interest (age, MDS–UPDRS III, LEDD, PD–CRS total score, prevalence of RBD, and NMSS total score). The analyses revealed that age [Odds Ratio (OR) 1.1, *p* = 0.001], MDS–UPDRS III (OR 1.1, *p* = 0.008), and RBD (OR 2.6, *p* = 0.011) were independent predictors of a more pronounced cognitive progression (Table 4).

**Table 4:** Binary logistic regression (progressors vs non-progressors)

	OR (95% CI)	*p* value
Age	1.09 (1.04–1.14)	0.001
MDS–UPDRS III	1.05 (1.01–1.08)	0.008
RBD	2.55 (1.24–5.25)	0.010

*OR* Odds ratio; *CI* confidence interval; *MDS–UPDRS* Movement Disorders Society–Unified PD Rating Scale; *RBD* REM–sleep behavior disorder

## Discussion

In this study we conducted a longitudinal analysis of 198 patients with PD–SCD which is, to our knowledge, the largest longitudinal cohort of PD–SCD subjects in whom cognitive evolution and the clinical variables involved in cognitive prognosis have been studied. The main results of the analysis showed that: (a) cognitive decline in PD–SCD is variable across individuals with either stability or progression in the long term, and loss of 16 points was considered significant; and (b) age, MDS–UPDRS III and RBD at baseline are independent predictors of a worse cognitive progression.

The prevalence of cognitive complaints in our cohort was 60.7% (198 out of 326), significantly higher than the previously estimated 36% in a recent meta-analysis [6]. However, our data falls within the range observed in previous studies (6.3–82.9%) [9]. These differences could be due to the scales used to assess both cognitive complaints (NMSS domain 5) or cognitive status (PD–CRS). Notably, the same meta-analysis found significantly lower rates of complaints in subjects with cognitive impairment compared to those without, suggesting that reduced insight and/or increased focus on motor or other NMS may contribute to this discrepancy in cognitively impaired PwP with more advanced PD. Our cohort includes relatively early and cognitively preserved PwP, thereby giving greater reliability to the complaints reported by the participants.

Most studies have indicated that cognitive complaints predict the development of cognitive impairment [6]. Nonetheless, cognitive decline is not uniform among PwP and thus, it is likely that not every PD–SCD patient follow the same pattern of progression [1]. To explore the differences between those experiencing more and less severe decline, we performed a *data-driven* approach to divide our PD–SCD cohort according to cognitive progression using the RCI for the PD–CRS, an instrument specifically validated for the assessment of cognitive status in PD [4, 19]. At baseline, PD–SCD Progressors were slightly older, presented more severe motor disease and more NMS, including higher rates of RBD. The LME model showed that the cognitive trajectory of these subgroups are significantly different with an estimated loss of 26.9 points in the PD–CRS total score at year 4, which is a clinically significant change [14]. Similarly, the proportion of PD–MCI in the long term was significantly higher in Progressors. However, there were no differences in terms of motor trajectories, despite significant MDS–UPDRS III differences at baseline, suggesting that the more aggressive cognitive phenotype is not necessarily associated with a similarly aggressive motor phenotype.

Data on the relationship between specific cognitive complaints and future cognitive impairment is scarce and has yielded varied results. Our data indicate that memory but not concentration/attention-related complaints, are associated with worse cognitive decline. Conversely, other studies have reported that complaints in executive abilities rather than those related to memory, attention or decision-making, are associated with cognitive decline [20]. A recent study by Weintraub et al. demonstrated that complaints in executive abilities, followed by those related to memory and cognitive slowing are the most frequently associated with future cognitive impairment [21]. Overall, these findings suggest that a broad range of complaints can serve as predictors of cognitive decline. Therefore, in both clinical and research settings, a comprehensive assessment of cognitive complaints across all cognitive domains is essential. This can be appropriately achieved by utilizing broader scales, such as the MDS–Non Motor Scale (MDS–NMS), which it specifically assesses the subjective perception of difficulties in attention, executive functions, memory, language, and visuospatial domains [22].

Neuropsychiatric manifestations such as depression, apathy or visual hallucinations have also been associated with an increased risk of cognitive impairment in PD [23, 24]. In the present study, the severity of depressive symptoms was not sufficient to classify participants as having depression. Similarly, the data suggest that mood-related symptoms, which have been previously associated with PD–SCD, are not reliable indicators of a more aggressive cognitive progression in PD-SCD individuals. Visual hallucinations were reported in 30.3% of the cohort, which falls within the previously reported prevalence in early stage PD [25]. However, similar to mood-related symptoms, they were not associated with a more aggressive phenotype in PD–SCD. Given the consistent association between hallucinations and cognitive decline, further longitudinal studies are needed to determine whether these results are due to sample size, assessment methods, or if baseline visual hallucinations simply do not provide additional information about cognitive decline in PD–SCD [24].

The association between age and more severe motor disease with cognitive decline is well documented [26]. In our analysis, the presence of RBD also emerged as an independent risk factor for more pronounced cognitive decline in PD–SCD. Besides the specific pathologic changes subserving RBD and cognitive decline in PD, one can hypothesize a common neurotransmitter link, mainly cholinergic, gathering these symptoms [27, 28]. RBD is believed to stem from the dysfunction of pedunculopontine nucleus–laterodorsal tegmental complex cholinergic neurons [29]. Most cholinergic projections in the central nervous system originate from the basal forebrain and mesopontine tegmental area connecting with the brainstem, striatum, thalamus, hypothalamus, and cortex [27]. Cholinergic dysfunction in PD has been associated not only with RBD and cognitive impairment, but also with hallucinations, gait impairment, hyposmia, and other neuropsychiatric and autonomic symptoms [29]. Our results align with the suggested existence of a *cholinergic phenotype* in PD, characterized by a more malignant and diffuse disease [29, 30]. Although RBD has been previously linked to cognitive impairment in PD, it is relevant identifying these progression risk factors in selected patients with PD–SCD, as this population represents an ideal target for future clinical trials [31].

This study is not devoid of some limitations. On the one hand, the definition used for PD–SCD was based on NMSS domain 5 [15]. We acknowledge that this is a rater-administered instrument and, unlike other comprehensive instruments such as the MDS–NMS, it does not cover the entire spectrum of cognitive problems that may occur in PD [3]. However, it might be a reliable tool in the absence of validated methods for assessing the presence of cognitive complaints in PD, and has been previously used in other studies [10]. On the other hand, RBD was not assessed using polysomnography or a validated tool. Nonetheless, the clinical interview conducted by a movement disorders specialist familiarized with RBD could suffice for the clinical approach of the study. The study also has strengths to consider, such as the sample size and the clinical follow-up over a 4-year period. In addition, we used PD–CRS for cognitive assessment, a PD-validated tool with a broad scoring range that allows for the identification of subtle progressions over time. While these aspects could have been explored in greater depth, they constitute one of the most extensive longitudinal studies carried out on PD–SCD to date, and its results can be informative for the design of future studies.

In summary, our results indicate that PD–SCD is frequent, but its presence can have different prognostic implications. Subjects with PD–SCD do not consistently show cognitive decline, but rather a variable cognitive progression. Age, MDS–UPDRS III and RBD at baseline significantly increase the risk of developing a more aggressive cognitive phenotype over a 4-year period. These findings are clinically relevant, and they should be considered for the characterization and prognosis of PwP. At the same time, they provide new insights into PD–SCD and how to study preclinical forms of cognitive impairment in PD. Future research incorporating ancillary tests such as fluid, genetic, neuroimaging, or neurophysiological measures will help explore additional interactions and prognostic markers in PD–SCD.
