# Correction: Heterogeneity of cognitive progression and clinical predictors in Parkinson’s disease–subjective cognitive decline

**DOI:** 10.1007/s00415-025-13077-1

**Published:** 2025-04-26

**Authors:** Jon Rodríguez-Antigüedad, Saül Martínez-Horta, Arnau Puig-Davi, Andrea Horta-Barba, Javier Pagonabarraga, Teresa de Deus Fonticoba, Silvia Jesús, Marina Cosgaya, Juan García Caldentey, María Asunción Ávila-Rivera, Nuria Caballol, Inés Legarda, Jorge Hernández Vara, Iria Cabo, Lydia López Manzanares, Isabel González Aramburu, Víctor Gómez Mayordomo, Jessica González Ardura, Julio Dotor García-Soto, Carmen Borrué, Berta Solano Vila, María Álvarez Sauco, Lydia Vela, Sonia Escalante, Esther Cubo, Zebenzui Mendoza, Isabel Pareés, Pilar Sánchez Alonso, María G. Alonso Losada, Nuria López Ariztegui, Itziar Gastón, Javier Ruíz Martínez, María Teresa Buongiorno, Carlos Ordás, Caridad Valero, Víctor Puente, Mónica Kurtis, Marta Blázquez Estrada, Pablo Martínez-Martín, Pablo Mir, Diego Santos-García, COPPADIS Study Group, A. D. Adarmes, M. Almeria, M. G. Alonso Losada, A. Alonso Cánovas, F. Alonso Frech, R. Alonso Redondo, I. Álvarez, M. Álvarez Sauco, A. Aneiros Díaz, S. Arnáiz, S. Arribas, A. Ascunce Vidondo, M. Aguilar, M. A. Ávila, N. Bernardo Lambrich, H. Bejr-Kasem, M. Blázquez Estrada, M. Botí, C. Borrue, M. T. Buongiorno, C. Cabello González, I. Cabo López, N. Caballol, A. Cámara Lorenzo, H. Canfield Medina, E. Carabajal Pendón, F. Carrillo, F. J. Carrillo Padilla, E. Casas, M. J. Catalán, P. Clavero, A. Cortina Fernández, M. Cosgaya, A. Cots Foraster, A. Crespo Cuevas, E. Cubo, T. de Deus Fonticoba, O. de Fábregues-Boixar, M. Díez-Fairen, J. Dotor García-Soto, E. Erro, S. Escalante, E. Estelrich Peyret, N. Fernández Guillán, P. Gámez, M. Gallego, J. García Caldentey, C. García Campos, C. García Díez, J. M. García Moreno, I. Gastón, M. P. Gómez Garre, V. Gómez Mayordomo, J. González Aloy, I. González-Aramburu, J. González Ardura, B. González García, M. J. González Palmás, G. R. González Toledo, A. Golpe Díaz, M. Grau Solá, G. Guardia, J. Hernández Vara, A. Horta-Barba, D. Idoate Calderón, J. Infante, S. Jesús, J. Kulisevsky, M. Kurtis, C. Labandeira, M. A. Labrador, F. Lacruz, M. Lage Castro, S. Lastres Gómez, I. Legarda, N. López Ariztegui, L. M. López Díaz, D. López Domínguez, L. López Manzanares, B. López Seoane, S. Lucas del Pozo, Y. Macías, M. Mata, G. Martí Andres, M. J. Martí, J. C. Martínez Castrillo, P. Martinez-Martin, D. McAfee, M. T. Meitín, Z. Mendoza Plasencia, M. Menéndez González, C. Méndez del Barrio, P. Mir, J. Miranda Santiago, M. I. Morales Casado, A. Moreno Diéguez, I. Muro García, V. Nogueira, A. Novo Amado, S. Novo Ponte, C. Ordás, J. Pagonabarraga, I. Pareés, B. Pascual-Sedano, P. Pastor, A. Pérez Fuertes, R. Pérez Noguera, A. Planas-Ballvé, L. Planellas, M. A. Prats, C. Prieto Jurczynska, V. Puente, M. Pueyo Morlans, A. Puig Daví, N. Redondo Rafales, L. Rodríguez Méndez, A. B. Rodríguez Pérez, F. Roldán, M. Ruíz De Arcos, J. Ruíz Martínez, P. Sánchez Alonso, M. Sánchez-Carpintero, G. Sánchez Díez, A. Sánchez Rodríguez, P. Santacruz, D. Santos García, J. C. Segundo Rodríguez, M. Seijo, M. Sierra Peña, B. Solano Vila, E. Suárez Castro, J. P. Tartari, C. Valero, L. Vargas, L. Vela, C. Villanueva, B. Vives, Jaime Kulisevsky

**Affiliations:** 1 Medicine Department, Universitat Autònoma de Barcelona (UAB), Barcelona, Spain; 2 Movement Disorders Unit, Neurology Department, Hospital de la Santa Creu i Sant Pau, Mas Casanovas 90, 08041 Barcelona, Spain; 3 Institut d´Investigacions Biomèdiques-Sant Pau (IIB-Sant Pau), Barcelona, Spain; 4 Centro de Investigación Biomédica en Red-Enfermedades Neurodegenerativas (CIBERNED), Madrid, Spain; 5 Neurology Department, Complejo Hospitalario Universitario de Ferrol (CHUF), A Coruña, Spain; 6 Movement Disorders Unit, Neurology and Clinical Neurophysiology Department, Instituto de Biomedicina de Sevilla, Hospital Universitario Virgen del Rocío/CSIC, Universidad de Sevilla, Seville, Spain; 7 Neurology Department, Hospital Clínic de Barcelona, Barcelona, Spain; 8 Centro Neurológico Oms 42, Neurology Department, Palma, Spain; 9 Neurology Department, Consorci Sanitari Integral, Hospital Moisés Broggi Sant Joan Despí, Barcelona, Spain; 10 Neurology Department, Hospital Universitario Son Espases, Palma, Spain; 11 Neurology Department, Hospital Universitario Vall d´Hebron, Barcelona, Spain; 12 Neurology Department, Complejo Hospitalario Universitario de Pontevedra (CHOP), Pontevedra, Spain; 13 Neurology Department, Hospital Universitario La Princesa, Madrid, Spain; 14 Neurology Department, Hospital Universitario Marqués de Valdecilla-IDIVAL, Santander, Spain; 15 Neurology Department, Institute of Neuroscience, Vithas Madrid La Milagrosa University Hospital, Vithas Hospital Group, Madrid, Spain; 16 Neurology Department, Hospital de Cabueñes, Gijón, Spain; 17 Neurology Department, Hospital Universitario Virgen Macarena, Seville, Spain; 18 Neurology Department, Hospital Infanta Sofía, Madrid, Spain; 19 Neurology Department, Institut d’Assistència Sanitària (IAS), Institut Català de la Salut, Girona, Spain; 20 Neurology Department, Hospital General Universitario de Elche, Elche, Spain; 21 Neurology Department, Fundación Hospital de Alcorcón, Madrid, Spain; 22 Neurology Department, Hospital de Tortosa Verge de la Cinta (HTVC), Tortosa, Tarragona Spain; 23 Neurology Department, Complejo Asistencial Universitario de Burgos, Burgos, Spain; 24 Neurology Department, Hospital Universitario de Canarias, San Cristóbal de la Laguna, Santa Cruz de Tenerife, Spain; 25 Neurology Department, Hospital Universitario Ramón y Cajal, IRYCIS, Madrid, Spain; 26 Neurology Department, Hospital Universitario Puerta de Hierro, Madrid, Spain; 27 Neurology Department, Hospital Álvaro Cunqueiro, Complejo Hospitalario Universitario de Vigo (CHUVI), Vigo, Spain; 28 Neurology Department, Complejo Hospitalario de Toledo, Toledo, Spain; 29 Neurology Department, Complejo Hospitalario de Navarra, Pamplona, Spain; 30 Neurology Department, Hospital Universitario Donostia, Donostia, Spain; 31 Neurology Department, Hospital Universitari Mutua de Terrassa, Terrassa, Barcelona Spain; 32 Neurology Department, Hospital Rey Juan Carlos, Madrid, Spain; 33 Neurology Department, Hospital Arnau de Vilanova, Valencia, Spain; 34 Neurology Department, Hospital del Mar, Barcelona, Spain; 35 Neurology Department, Hospital Ruber Internacional, Madrid, Spain; 36 Neurology Department, Hospital Universitario Central de Asturias, Oviedo, Spain; 37 Neurology Department, Complejo Hospitalario Universitario de A Coruña (CHUAC), INIBIC, A Coruña, Spain; 38 Fundación Española de Ayuda a La Investigación en Enfermedades Neurodegenerativas y/o de Origen Genético, Oleiros, Spain

**Correction: Journal of Neurology (2025) 272:246** 10.1007/s00415-024-12808-0

In the original version of this article, abstract was missing and should have read

**Background** Parkinson’s Disease (PD)-associated subjective cognitive decline (PDSCD) is defined as cognitive complaints without objective cognitive impairment. Based on most studies, it is associated with a greater risk of cognitive decline and may represent a prodromal stage of cognitive impairment.

**Methods** The main objectives are to identify cognitive progression patterns and clinical predictors of worse cognitive decline within a large PD-SCD cohort with a 4-year follow-up. All patients belong to the prospective observational multicenter study COPPADIS.

**Results** A total of 198 PD-SCD subjects were analyzed. Mean age was 60.9, mean disease duration 5.2, and mean PD-Cognitive Rating Scale (PD-CRS) 97.6. Subjects were classified as Progressors if their Reliable Change Index was ≤ − 1.64 at year 4, and as non-Progressors if it was > − 1.64 (− 1.64 corresponded to − 16 on the PD-CRS). Progressors had significantly higher age, Movement Disorders Society-Unified PD Rating Scale (MDS-UPDRS) III, levodopa equivalent daily-dose, Non-Motor Symptom Scale total score, memory-related cognitive complaints, and prevalence of REM-sleep behavior disorder (RBD) at baseline. A linear mixed-effects model showed divergent cognitive trajectories between Progressors and non-Progressors (estimate = − 26.8; p < 0.001), with no differences in motor trajectories. In the binary regression model, age (OR = 1.09; p = 0.001), MDS-UPDRS III (OR = 1.05, p = 0.008), and RBD (OR = 2.55, p = 0.010) at baseline were independent predictors of cognitive progression.

**Conclusions** Subjects with PD-SCD do not consistently show cognitive decline, but rather exhibit a heterogeneous progression. Age, MDS-UPDRS III and RBD significantly increase the risk of a more aggressive cognitive phenotype. Future research on biomarkers will help explore additional cognitive predictors in PD-SCD.

The original article has been corrected.

